# Testosterone promotes the migration, invasion and EMT process of papillary thyroid carcinoma by up-regulating Tnnt1

**DOI:** 10.1007/s40618-023-02132-1

**Published:** 2023-07-21

**Authors:** C. Jiang, F. Xu, D. Yi, B. Jiang, R. Wang, L. Wu, H. Ding, J. Qin, Y. Lee, J. Sang, X. Shi, L. Su

**Affiliations:** 1 Division of Thyroid Surgery, Department of General Surgery, Nanjing Drum Tower Hospital, the Affiliated Hospital of Medical School, Nanjing University, Nanjing, 210008 Jiangsu China; 2 Department of General Surgery, Nanjing Drum Tower Hospital, Clinical College of Nanjing Medical University, Nanjing, 221000 Jiangsu China

**Keywords:** Papillary thyroid carcinoma, RNA sequencing, Testosterone, Tnnt1, p38/JNK signaling pathway

## Abstract

**Purpose:**

To explore the key genes and molecular pathways in the progression of thyroid papillary carcinoma (PTC) promoted by testosterone using RNA-sequencing technology, and to provide new drug targets for improving the therapeutic effect of PTC.

**Methods:**

Orchiectomy (ORX) was carried out to construct ORX mouse models. TPC-1 cells were subcutaneously injected for PTC formation in mice, and the tumor tissues were collected for RNA-seq. The key genes were screened by bioinformatics technology. Tnnt1 expression in PTC cells was knocked down or overexpressed by transfection. Cell counting kit-8 (CCK-8), colony formation assay, scratch assay and transwell assay were adopted, respectively, for the detection of cell proliferation, colony formation, migration and invasion. Besides, quantification real-time polymerase chain reaction (qRT-PCR) and western blot were utilized to determine the mRNA and protein expression levels of genes in tissues or cells.

**Results:**

Both estradiol and testosterone promoted the growth of PTC xenografts. The key gene Tnnt1 was screened and obtained by bioinformatics technology. Functional analysis revealed that overexpression of Tnnt1 could markedly promote the proliferation, colony formation, migration, invasion, and epithelial-to-mesenchymal transition (EMT) process of PTC cells, as well as could activate p38/JNK pathway. In addition, si-Tnt1 was able to inhibit the cancer-promoting effect of testosterone.

**Conclusion:**

Based on the outcomes of bioinformatics and basic experiments, it is found that testosterone can promote malignant behaviors such as growth, migration, invasion and EMT process of PTC by up-regulating Tnnt1 expression. In addition, the function of testosterone may be achieved by activating p38/JNK signaling pathway.

**Supplementary Information:**

The online version contains supplementary material available at 10.1007/s40618-023-02132-1.

## Introduction

Thyroid carcinoma (TC) has seen a rapid rise in incidence over the past few decades, and it is predicted to be the fourth most common cancer by 2030 [1]. On basis of the origin and growth pattern of cell types, TC has been divided into the following four types: differentiated papillary carcinoma, follicular carcinoma, medullary carcinoma, and undifferentiated/anaplastic carcinoma [2]. Among them, papillary thyroid carcinoma (PTC) is the most common TC, accounting for 85% of all TCs, and its incidence has been climbing up year by year [3]. Even though considerable therapeutic efficacy has been achieved, PTC is still worrisome in terms of its high prevalence. More terribly, its recurrence rate and cause-specific mortality nearly have approached 30% and 8.6% in the past 30 years, respectively [4]. Therefore, it is an urgent and necessary task to find diagnostic markers and new therapeutic targets for the recurrence and metastasis of PTC. Notably, researchers have found notable gender differences in the incidence of PTC as it rises. Specifically, PTC is the 7th most common malignancy in women, but it is not one of the 15 most common cancers in men; and it is reported that the incidence of PTC in women is almost three times that in men [5].

Sex hormone is an important contributor to the sex difference in PTC incidence. Especially, the effect of estrogen on TC has been widely studied in recent years [6]. Unlike estrogen, the effect of androgens on TC remains controversial. Testosterone is an androgen hormone that is secreted mainly by the male testis or female ovaries, and can also produce by the adrenal glands [7]. Testosterone has multiple physiological functions. However, recent studies show that the expression level of testosterone is highly associated with the occurrence, development, and prognosis of various diseases, including cardiovascular disease, depression, obesity, and prostate cancer [8–10]. For example, Kumar and Banu et al. pointed out that estradiol and testosterone could promote the growth and metastasis of PTC [11, 12]. But their mechanism of action is still unclear, and further exploration is required. Androgens and thyroid hormone (TH) may interact and mediate coordinated effects on human prostate cancer formation and progression by playing proliferative and pro-inflammatory roles [13]. Some studies have reported that androgen could activate epithelial-to-mesenchymal transition (EMT) and its effectors, while others have shown EMT activation caused by androgen signaling inhibition [14, 15]. Testosterone, and its prostate metabolite dihydrotestosterone (DHT), induce cell proliferation, tumor growth, and probably, metastasis [16]. According to previous studies, DHT treatment can decrease epithelial expression of E-cadherin and β-catenin but increase the expression of the mesenchymal marker proteins Vimentin and N-cadherin [17]. EMT, a cell biological process involving wound healing and embryogenesis, is often aberrantly activated in cancer and can promote migration, invasion, and metastasis [18, 19]. The inhibition of the EMT progression is essential for the treatment of PTC.

Whole transcriptome analysis including transcriptome sequencing and bioinformatics can reveal differentially expressed genes (DEGs) in PTC tissues with or without sex hormone stimulation [20]. Genetic alterations in any cancers (including PTC) can reflect the biodiversity of cellular phenotypes and physiological functions. These alterations may become crucial study topics for understanding the molecular mechanisms by which estradiol and testosterone promote PTC progression. Therefore, in order to clarify the mechanism of androgen in PTC, we constructed orchiectomy (ORX) mouse models and explored the roles of estradiol and testosterone in PTC growth by injecting estradiol and testosterone into the mice. Subsequently, a series of RNA sequencing and bioinformatics analysis were adopted to screen DEGs in testosterone-treated tumors. Besides, functional annotation and pathway enrichment of DEGs, and protein–protein interaction (PPI) network construction were performed in testosterone-treated tumors. These regulatory networks made it possible to identify genes with key roles in PPI networks and to further clarify whether testosterone exerted its functions in the progression of PTC through those key genes.

## Materials and methods

### Orchiectomy mouse model construction and group treatment

**Fig. 1:**
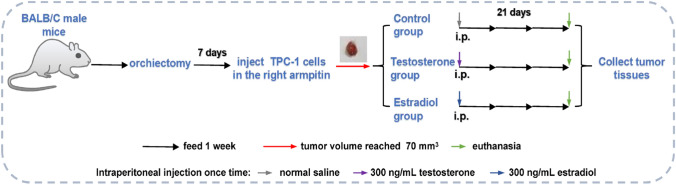
A flow chart of mouse model construction and group treatment

A total of 9 BALB/C male mice were selected for 1-week adaptive feeding, then they were subjected to ORX. Specifically, the mice were first anesthetized with 2% sodium pentobarbital for following ORX; second, midline incisions were made in the midline of the scrotum and the lower capsule, respectively, to expose the testis; finally, the vas deferens and spermatic vessels were cauterized, and the incisions were sutured [21]. One week after recovery, the ORX mice were subcutaneously injected with 4 × 10^6^ PTC cell line TPC-1 in the right armpit. After the tumor volume reached 70 mm^3^, the mice were randomly divided into three groups (*n* = 3). To be specific, mice were intraperitoneally injected with normal saline (Control group), 300 ng/mL testosterone (Testosterone group) and 300 ng/mL estradiol (Estradiol group) once time, respectively. Following 21 days of feeding, the mice were euthanized, and their tumor tissues were collected. The flow chart of the experiment is shown in Fig. 1. This study was approved by the Experimental Animal Ethics Committee of Nanjing Drum Tower Hospital (2022AE01002), and all experiments were conducted in accordance with the approved guidelines.

### RNA sequencing

After the mice were euthanized, 100 mg of tumor tissues was collected from three mice of each group. Then, their total RNA was extracted using an RNA extraction kit following the manufacturer’s instructions. The quality and quantity of the extracted RNA were determined using a Nanodrop spectrophotometer and an Agilent 2100 Bioanalyzer, respectively. RNA libraries were prepared from 250 ng of total RNA using the Illumina Exome Capture Kit. RNA sequencing was performed following the standard protocol of the DFCI Molecular Biology Core Facility (Illumina NextSeq 500).

### Screening of differentially expressed genes (DEGs)

FPKM (Fragments Per Kilobase of exon model per Million mapped reads) was used to measure the abundance value of gene expression. The expression abundance of known genes in different samples was statistically analyzed by FPKM. The result of FPKM was equivalent to the expression of genes in different samples. |log (fold change (FC))|≥ 1 and *P* < 0.05 were determined as the basic conditions for screening differentially expressed genes (DEGs). The R package DESeq2 was applied for screening DEGs, Enhanced Volcano for plotting the volcano plots.

### Gene ontology (GO) enrichment analysis

The basic standards for screening DEGs were determined as |log (FC) |≥ 1 and *P* < 0.05. Gene ontology (GO) analysis was performed to functionally enrich DEGs in accordance with cellular component (CC), biological process (BP), and molecular function (MF) based on GO database. Kyoto Encyclopedia of Genes and Genomes (KEGG) analysis, a biological pathway analysis for DEGs, was applied to identify the metabolic pathways and signal transduction pathways that DEGs involved in as well as to count the significance level of gene enrichment in the pathways. DAVID (https://david.ncifcrf.gov/) online database was employed to perform the above GO functional and KEGG pathway enrichment analyses of DEGs [22].

### Protein–protein interaction (PPI) analysis

The STRING interaction (https://string-db.org/) database website was adopted to build PPI networks based on gene overlap; besides, this website also could import obtained gene data into Cytoscape for subsequent analysis based on the MCC algorithm. Confidence (*C*) ≥ 0.7 was used as the cutoff criterion. Then, molecular complex detection (MCODE) was performed to filter the maximum PPI network modules with degree cutoff = 2, node score cutoff = 0.2, k-core = 2 [23].

### Cell culture and treatment

PTC cell lines (B-CPAP and TPC-1) were purchased from the National Collection of Authenticated Cell Cultures. The cells were cultured in RPMI-1640 medium containing 10% fetal bovine serum (FBS, Gibco) and 1% penicillin–streptomycin, and then placed in a cell incubator with 5% CO_2_ at 37 °C. Then B-CPAP and TPC-1 cells were seeded in 6-well plates and cultured to a confluence of about 80%. Later, negative pcDNA3.1 (NC), pcDNA3.1 Tnnt1 (Tnnt1) as well as negative siRNA (siNC) and Tnnt1 siRNA (si-Tnnt1) (Shanghai Genechem Co., LTD.) were transfected into B-CPAP and TPC-1 cells according to the instructions of Lipofectamine ™ 2000. The transfected cells were named as NC group, Tnnt1 group, siNC, and si-Tnnt1 group, respectively. In addition, in order to verify the relationship between testosterone and Tnnt1, those cells were first intervened using testosterone and then received transfection after 48 h of the intervention.

### Quantification real-time polymerase chain reaction (qRT-PCR)

After transfection, TRizol method was adopted for the extraction of the total RNA from cells, NanoDrop for the determination of RNA concentration and purity, and the Random Primer Reverse Transcription Kit (Thermo, USA) for the preparation of cDNA. In addition, the expression levels of the genes were detected according to the instructions of the SYBR GREEN kit (TaKaRa, Japan). The primer sequences are shown in Table 1. The relative expression level of the corresponding genes was calculated using GAPDH as an internal control. The experimental data obtained by quantification real-time polymerase chain reaction (qRT-PCR) were used to calculate the relative expression of the target genes using the $2^{{ - \Delta \Delta C_{{\text{t}}} }}$ method. The experiments were replicates twice, and the number of cells of each group is three.

**Table 1:** Primers for quantification

Gene	Sequences (5′–3′)
Tnnt1	F: AACGCGAACGTCAGGCTAAGCT
	R: CAGGGAGAAACGACCTGGAG
GAPDH	F: CATCACTGCCACCCAGAAGACTG
	R: ATGCCAGTGAGCTTCCCGTTCAG

### Cell counting kit-8 (CCK-8)

After transfection, cells were collected and seeded in 96-well plates at 2 × 10^4^ cells/well. Six replicates were set in each group. The cell proliferation was measured at 0 h of cell adherence and 24 h of treatment or no treatment according to the Cell Counting Kit-8 (CCK-8) instructions. Briefly, 10 μL of CCK-8 solution (Solarbio) was added to each well, and the incubation was continued in a cell incubator for another 2 h. The optical density of each well at 450 nm was measured using a microplate reader. The experiments were replicates twice, and the number of cells of each group is three.

### Cell colony formation assay

After transfection, cells were inoculated in 6-well plates at 1000 cells/well, and 3 replicates was set up for each group. The 6-well plates were placed in a cell incubator for 12–15 days, and the culture medium was observed every 3 days. When macroscopic cell colonies were formed, the culturing was discontinued, and the culture medium was discarded. Then, the cells were fixed with 4% paraformaldehyde, stained with 0.1% crystal violet staining solution, and dried. Finally, colony formation of cells in each group was photographed and recorded. The experiments were replicated twice, and the number of cells of each group is three.

### Transwell

Matrigel (50 mg/L) was diluted according to 1:15 with serum-free medium, then 100 μL of diluted matrigel was added to the bottom membrane of upper chamber of the transwell. To be specific, the matrigel was evenly spread in the upper chamber surface and placed in a cell incubator for solidification. Subsequently, the cells after transfection were resuspended with serum-free medium. Later, the concentration of the cell suspension was adjusted to 5 × 10^5^ cells/mL, and 100 µL suspension was added to the upper chamber. In addition, 600 μL of culture medium containing 20% FBS was added to a 24-well plate, then the mixtures were cultured in a cell incubator for another 20–24 h. Next, the inserts were taken out, and the cells and gels did not cross the membrane in the upper chamber were absorbed. Later, 4% paraformaldehyde was applied for fixation, and crystal violet staining solution for staining. After being dried, the transwell inserts were observed and photographed under a microscope. The experiments were replicates twice, and the number of cells of each group is three.

### Scratch test

After transfection, cells were seeded into 12-well plates at 2 × 10^5^ cells/well and cultured to confluence, with 3 replicate wells for each group. Subsequently, the cell monolayer was scratched with a pipette tip, and the separated cells were removed by serum-free medium. Later, the cells were treated using corresponding testosterone, and the cell scratches of each group were observed and photographed at 0 h after scratching and at 24 h of culture after scratching. The experiments were replicated twice, and the number of cells of each group is three.

### Western blot

Total cellular proteins were extracted on ice using RIPA lysis solution (Solarbio), and the concentration of the extracted proteins was determined with a BCA kit (Beyotime). Next, 20 μg of proteins were boiled and denatured after adding 5 × loading buffer, then their separation was performed by SDS-PAGE electrophoresis. Later, the proteins were transferred to PVDF membranes, and 5% skimmed milk powder was added to the blocking solution to block the membranes for 2–3 h. After being washed by TBST for three times, primary antibodies (anti-MMP-2, anti-MMP-9, anti-E-cadherin, anti-Cacna1s, anti-p38, anti-p-p38, anti-JNK, anti-p-JNK, anti-β-actin, Abcam, USA) were added for incubation on a shaker overnight at 4 °C. After three TBST washes of the membranes again, secondary antibodies (ZSGB-BIO, ZB2301, ZB2305) were added for 1-h incubation at ambient temperature. Relative protein expression was calculated using β-actin as an internal control. The experiments were replicates twice, and the number of cells of each group is three.

### Tumor formation test

Fifteen 8-week-old BALB/C nude mice were randomly divided into three groups (siNC group, T + siNC, T + si-Tnnt1 group). In T + siNC and T + si-Tnnt1 groups, testosterone was placed under the skin tissues of the mice through subcutaneous implantation. Then, 4 × 10^6^ TPC-1 cells transfected with negative siNC or si-Tnt1 were subcutaneously injected into the right axilla of mice in siNC group, T + siNC T group and T + si-Tnnt1 group. Twenty-one days after cell injection, the mice were euthanized, and tumor tissues were weighed. After that, the length and width of the tumor were measured using a vernier caliper. The tumor volume was calculated by the following formula: tumor volume = (length × width^2^)/2.

### Statistical analysis

All experimental data in this study were expressed as mean ± standard deviation (SD). SPSS 17.0 software was used for statistical analysis. The distribution of continuous data was checked for normality using Shapiro–Wilk test. Independent sample *t* test was applied for comparison between two groups, and one-way analysis of variance followed by the Tukey post hoc tests for comparison between multiple groups.* P* < 0.05 was considered as the criterion for judging the significance of differences.

## Results

### Testosterone promotes the growth of mouse xenograft tumors and the screening of differentially expressed genes causing tumors

**Fig. 2:**
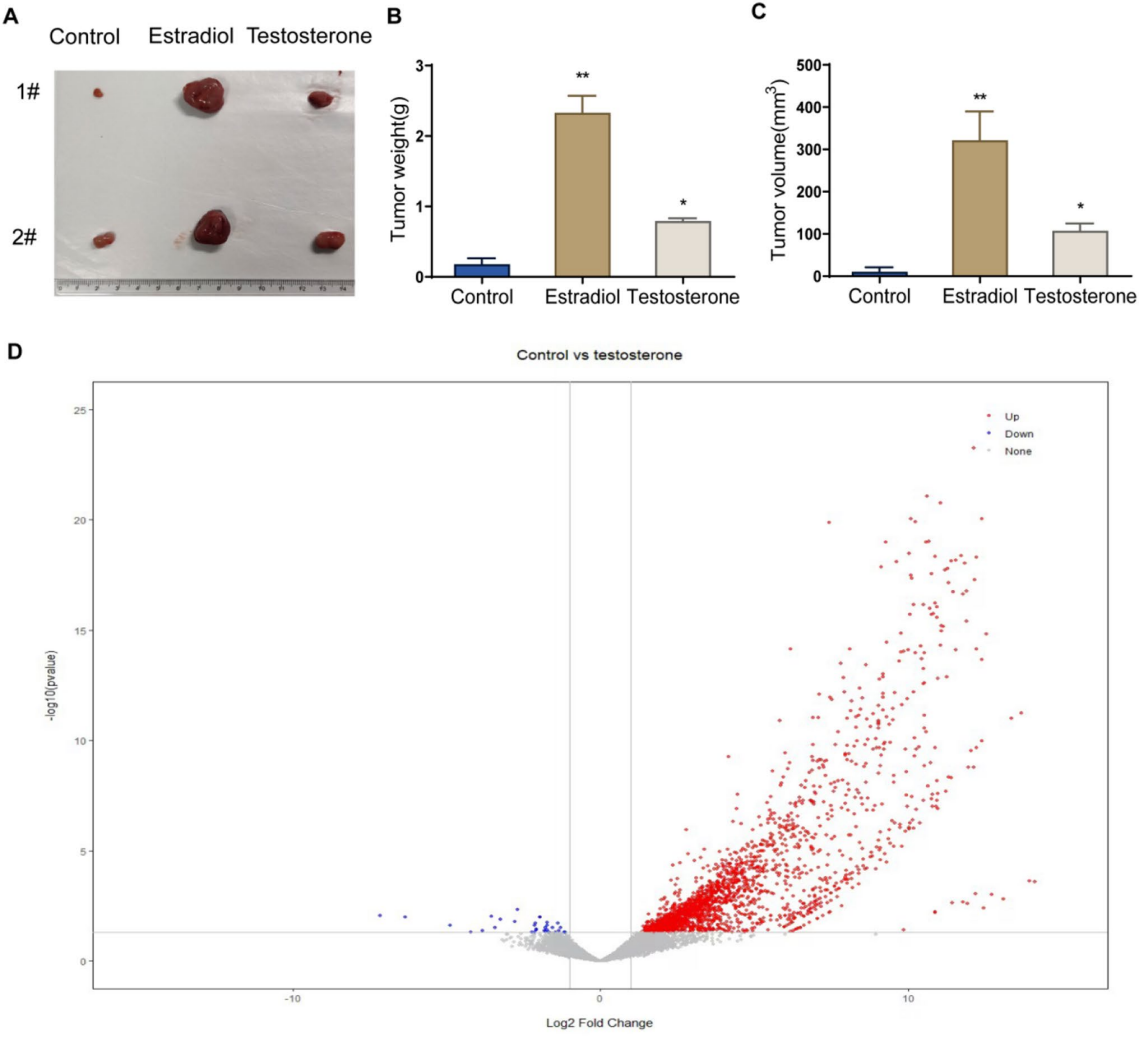
Effect of testosterone on gene expression in mouse xenograft tumors. The effect of testosterone on the extrinsic feature (**A**), weight (**B**) and volume (**C**) of xenograft formed by TPC-1 cells. **D** Differentially expressed genes (DEGs) in tumor tissues of mice in the testosterone group and control group visualized by volcano plot. **P < 0.01, vs. control, ^##^*P* < 0.01, vs. Estradiol

To explore the effect of testosterone or estradiol on the development of PTC, mouse models of PTC xenograft after ORX were constructed and injected with testosterone or estradiol. After 21 days, the tumor volume and weight of mice in the Testosterone and Estradiol group were significantly increased compared with those in the Control group, and the tumor growth rate of mice in the Testosterone group was significantly slower than that of mice in the Estradiol group (Fig. 2A–C). These results suggested that male hormones were able to promote the development of PTC.

To reveal the mechanism of action of testosterone in PTC, the tumor tissues of the testosterone and Control groups were collected for RNA sequencing, and then the DEGs were screened according to the standard of |log (fold change (FC))|≥ 1 and *P* < 0.05. Based on the results, 2042 genes showed a significant up-regulation trend, and the expression of 32 genes was significantly down-regulated in the tumor tissues of mice in the Testosterone group compared with the Control group (Fig. 2D), indicating the fact that testosterone treatment could change the gene expression in PTC tumors.

### Gene ontology (GO) analysis and Kyoto encyclopedia of genes and genomes (KEGG) pathway analysis of differentially expressed genes

**Fig. 3:**
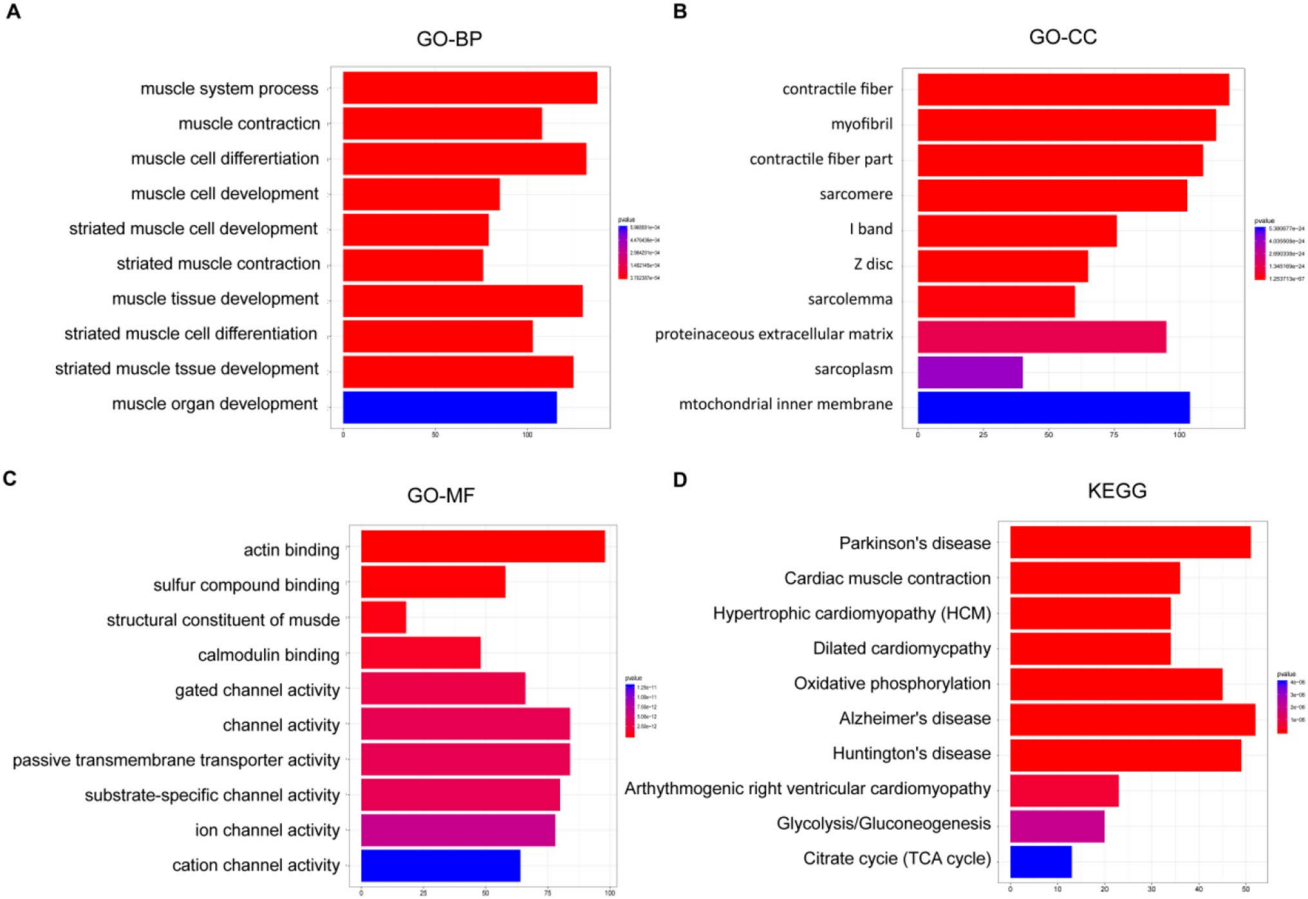
GO functional enrichment analysis and KEGG pathway analysis of DEGs. **A** DEGs were mainly enriched in related biological processes such as muscle system process, muscle cell differentiation, and striated muscle by GO analysis. **B** Enriched DEGs were mainly associated with cellular components such as contractile fiber, proteinaceous extracellular matrix, and sarcolemma based on GO analysis. **C** Enriched DEGs were mainly associated with molecular functions such as actin binding, sulfur channel binding, and contractile activity based on GO analysis. **D** KEGG analysis for the molecular biological pathways of DEGs enrichment mainly concentrated in the TCA cycle and oxidative phosphorylation pathway

In order to reveal the mechanism that testosterone promoted the progression of PTR, we performed GO functional enrichment analysis and KEGG pathway analysis of DEGs, respectively. The results of GO functional enrichment analysis showed that DEGs were mainly enriched in biological processes such as muscle system process, muscle cell differentiation, and striated muscle contraction (Fig. 3A–C). Besides, enriched DEGs were mainly associated with cellular components such as contractile fiber, proteinaceous extracellular matrix, and sarcolemma, as well as molecular functions such as actin binding, sulfur channel binding, and contractile activity. However, KEGG analysis presented that DEGs were mainly concentrated in the TCA cycle and oxidative phosphorylation pathways (Fig. 3D).

### Construction of protein–protein interaction (PPI) network

**Fig. 4:**
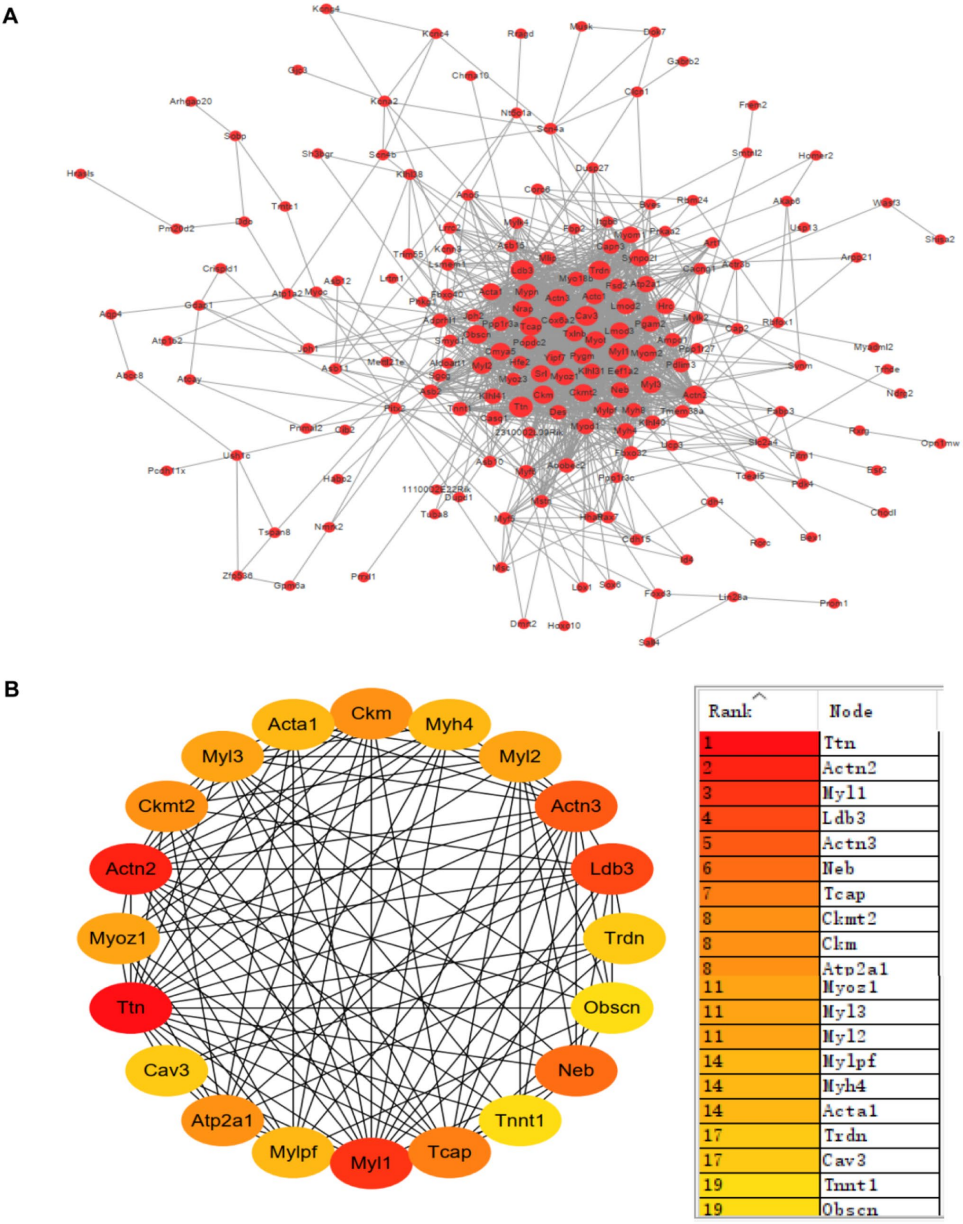
Protein–protein interaction (PPI) network construction. **A**, **B** PPI network of the top 20 key DEGs constructed by Cytoscape

Subsequently, to further investigate the function of DEGs, the screened DEGs with |log (fold change (FC)) |≥ 7 and *P* < 0.05 were selected to construct a PPI network (Fig. 4A), and the top 20 key Hub genes were searched by cytoHubba to construct a subnetwork (Fig. 4B). The ranking of key DEGs were Ttn, Actn2, Myl1, Ldb3, Actn3, Neb, Tcap, Atp2a1, Ckmt2, Ckm, Myl3, Myoz1, Myl2, Mylpf, Acta1, Myh4, Trdn, Cav3, Obscn, and Tnnt1.

### Tnnt1 promotes the proliferation and migration of papillary thyroid carcinoma (PTC) cells

**Fig. 5:**
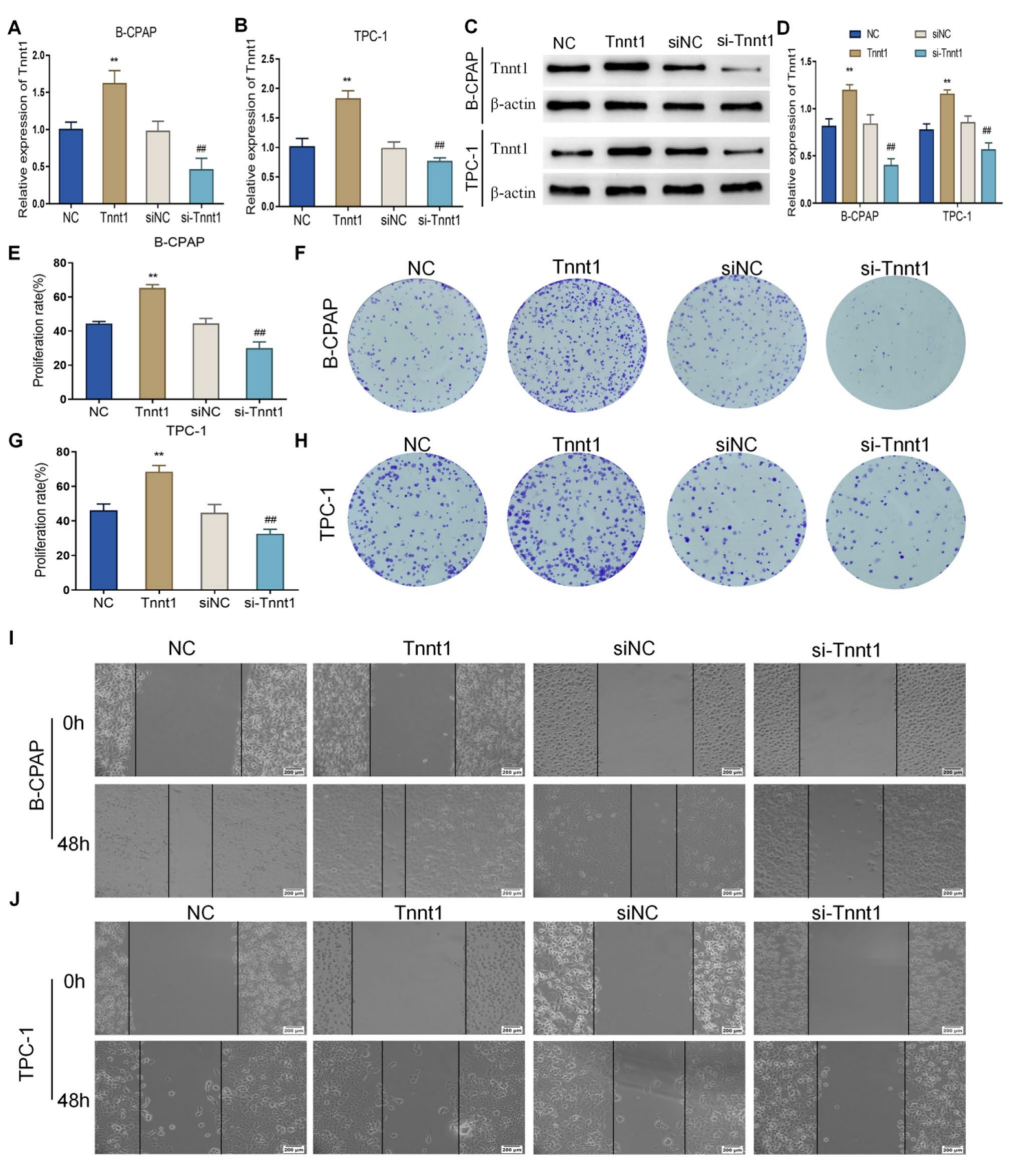
Tnnt1 promotes the proliferation and migration of papillary thyroid carcinoma (PTC) cells. The efficiency of Tnnt1 after transfection, overexpress or knockdown in B-CPAP (**A**) and TPC-1 (**B**) cells detected by qRT-PCR. **C**, **D** The efficiency of Tnnt1 after transfection, overexpress or knockdown in B-CPAP and TPC-1 cells detected by western blot. The proliferation of B-CPAP (**E**) and TPC-1 (**F**) cells after overexpression or knockdown of Tnnt1 detected by CCK-8. The colony formation of B-CPAP (**G**) and TPC-1 (**H**) cells after overexpression or knockdown of Tnnt1 observed by colony formation assay. The migration ability of B-CPAP (**I**) and TPC-1 (**J**) cells after overexpression or knockdown of Tnnt1 assessed by scratch assay, **P < 0.01, vs. NC, ^##^*P* < 0.01, vs. siNC

Based on the screening of key Hub genes in PPI network of DEGs and previous studies, we selected Tnnt1 for further functional analysis. According to the findings of RNA sequencing, Tnnt1 was significantly highly expressed in PTC tissues, so it could be speculated that Tnnt1 might be a key gene in the promotion of the growth and progression of PTC cells treated by testosterone. Subsequently, cell experiments were performed to explore the function of Tnnt1. First, Tnnt1 expression in B-CPAP and TPC-1 cells was knocked down or overexpressed through transfection (Fig. 5A–D), and then CCK-8 and cell colony formation assays were conducted. The outcomes exhibited that the proliferation rate and viability of cells in the Tnnt1 group were markedly increased compared with those in the NC group, whereas the proliferation rate and viability of cells in the si-Tnnt1 group were obviously decreased compared with those in the siNC group (Fig. 5E–H). The results of scratch assay showed that overexpression of Tnnt1 significantly enhanced the migration ability of PTC cells, while knockdown of Tnnt1 notably weakened PTC cells’ migration ability (Fig. 5I, J). Overall, Tnnt1 overexpression was able to significantly promote the proliferation and migration of PTC cells.

### Tnnt1 promotes invasion and epithelial-to-mesenchymal transition (EMT) progression in PTC cells

**Fig. 6:**
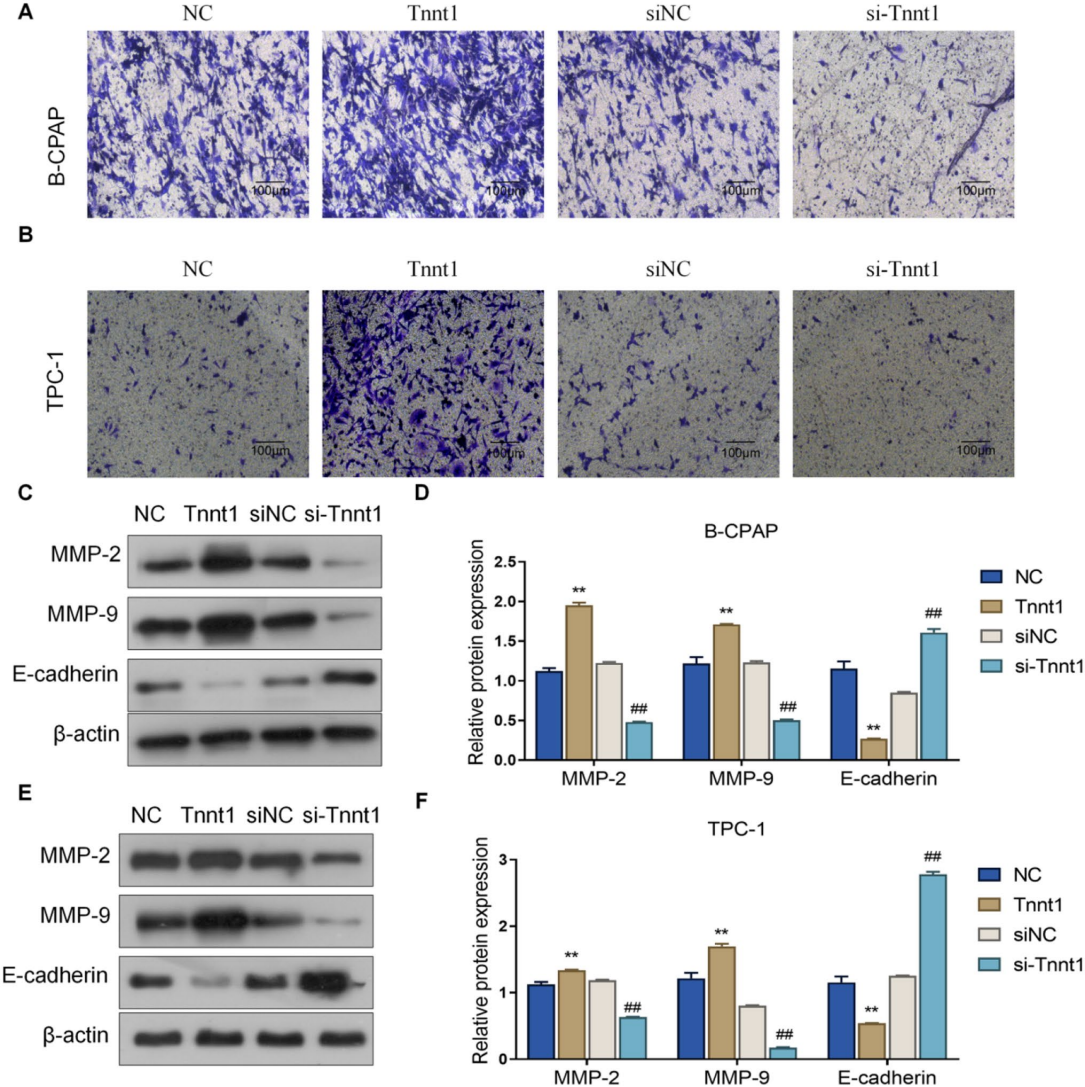
Tnnt1 promotes invasion and EMT progression in PTC cells. The invasive ability of B-CPAP (**A**) and TPC-1 (**B**) cells after overexpression or knockdown of Tnnt1 assessed by transwell assay; the expression of EMT markers MMP-2, MMP-9 and E-cadherin in B-CPAP (**C**, **D**) and TPC-1 (**E**, **F**) cells after overexpression or knockdown of Tnnt1 detected by western blot, **P < 0.01, vs. NC, ^##^*P* < 0.01, vs. siNC

Aside from the above, the effect of Tnnt1 on the invasion of PTC cells was also further explored by transwell assay, and the following results were obtained. First, the Tnnt1 group showed stronger invasion ability of B-CPAP and TPC-1 cells than the NC group, while that invasion ability was markedly lower in the si-Tnnt1 group than the siNC group (Fig. 6A, B). In addition, detection of expression of EMT progression markers (E-cadherin, MMP-2 and MMP-9) by western blot revealed that overexpression of Tnnt1 could significantly raise up the protein level of MMP-2 and MMP-9 while decreased the expression of E-cadherin in B-CPAP and TPC-1 cells. However, knockdown of Tnnt1 expression declined MMP-2 and MMP-9 protein level while increased E-cadherin (Fig. 6C–F). Taken together, Tnnt1 could promote the invasion and EMT process of PTC cells.

### Tnnt1 activates the p38/JNK signaling pathway in PTC cells

**Fig. 7:**
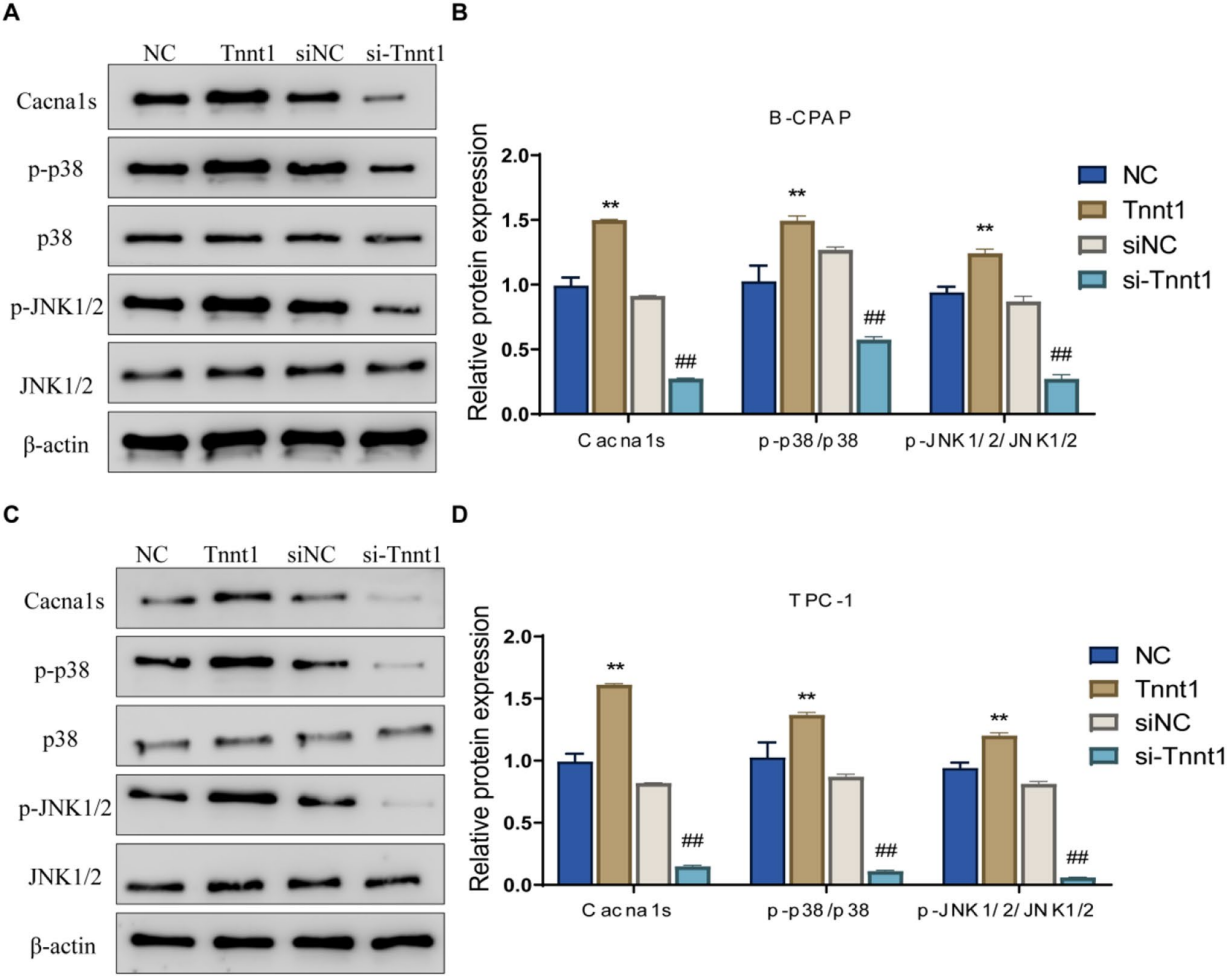
Tnnt1 activates the p38/JNK signaling pathway in PTC cells. The relative expression level of p38/JNK signaling pathway-related proteins (Cacnals, p-p38, p38, p-JNK, JNK) in B-CPAP (**A**, **B**) and TPC-1 (**C**, **D**) cells after overexpression or knockdown of Tnnt1 were detected by western blot, **P < 0.01, vs. NC, ^##^*P* < 0.01, vs. siNC

To figure out the molecular mechanism that Tnnt1 acted on PTC, the expression of p38/JNK pathway-related proteins was examined by western blot. In addition, the results showed that overexpression of Tnnt1 could obviously increase the protein levels of Cacnals, p-p38 and p-JNK and the ratios of p-p38/p38 and p-JNK/JNK in B-CPAP and TPC-1 cells. Knockdown of Tnnt1 expression, on the other hand, caused a significant decrease in the expression level of Cacnals and the ratios of p-p38/p38, p-JNK/JNK in B-CPAP and TPC-1 cells (Fig. 7A–D). Briefly speaking, Tnnt1 could activate the p38/JNK signaling pathway in a significant manner in PTC cells.

### Knockdown of Tnnt1 inhibits the promoting effect of testosterone on PTC development

**Fig. 8:**
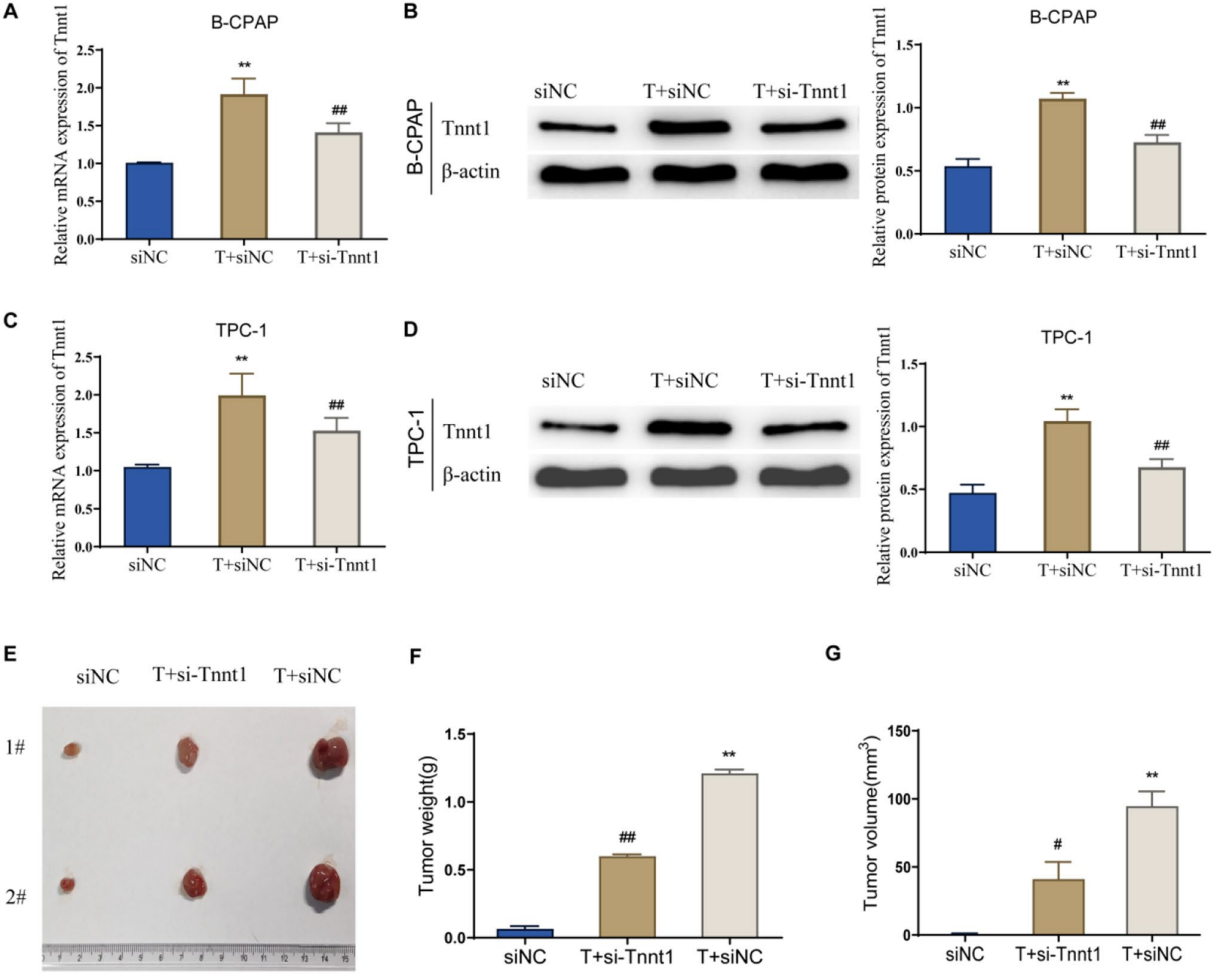
Knockdown of Tnnt1 expression can inhibit the promoting effect of testosterone on PTC development. **A**, **B** The mRNA and protein expression of Tnnt1 in B-CPAP cells after treatment with testosterone alone or that combined with knockdown of Tnnt1 expression observed by qRT-PCR and western blot. **C**, **D** The mRNA and protein expression of Tnnt1 in TCP-1 cells after treatment with testosterone alone or that combined with knockdown of Tnnt1 expression observed by qRT-PCR and western blot. **E** Tumor formation of TPC-1 cells treated with testosterone alone or that combined with knockdown of Tnnt1 expression in mice. Determination of weight (**F**) and volume (**G**) of xenografts formed by TPC cells treated with testosterone alone or that combined with knockdown of Tnnt1 expression in mice. **P < 0.01, vs. siNC, ^#^*P* < 0.05 and ^##^*P* < 0.01, vs*.* T + siNC

To further explore the roles of testosterone and Tnnt1, we knocked down Tnnt1 expression in B-CPAP and TPC-1 cells in addition to performing testosterone treatment. In addition, qRT-PCR results showed that Tnnt1 expression in PTC cells was significantly increased in the T + siNC group relative to that in the siNC group, while compared with the T + siNC group, Tnnt1 expression in cells in the T + si-Tnt1 group was significantly decreased (Fig. 8A–D). Then, testosterone was continuously put under the mice’ skin tissues by subcutaneous implantation, and si-Tnnt1-transfected TPC-1 cells were subcutaneously injected in the right axilla of the mice. The results disclosed that testosterone alone could significantly increase the weight and volume of xenograft tumors in siNC group mice; nevertheless, the size and volume of xenograft tumors in T + si-Tnt1 group mice were much smaller than those in the T + siNC group (*P* < 0.01) (Fig. 8E–G). The above outcomes suggested the fact that si-Tnt1 could significantly inhibit the testosterone-induced growth of PTC xenograft tumors.

### Testosterone plays a promoting role in the malignant behavior of PTC cells by up-regulating Tnnt1 expression

**Fig. 9:**
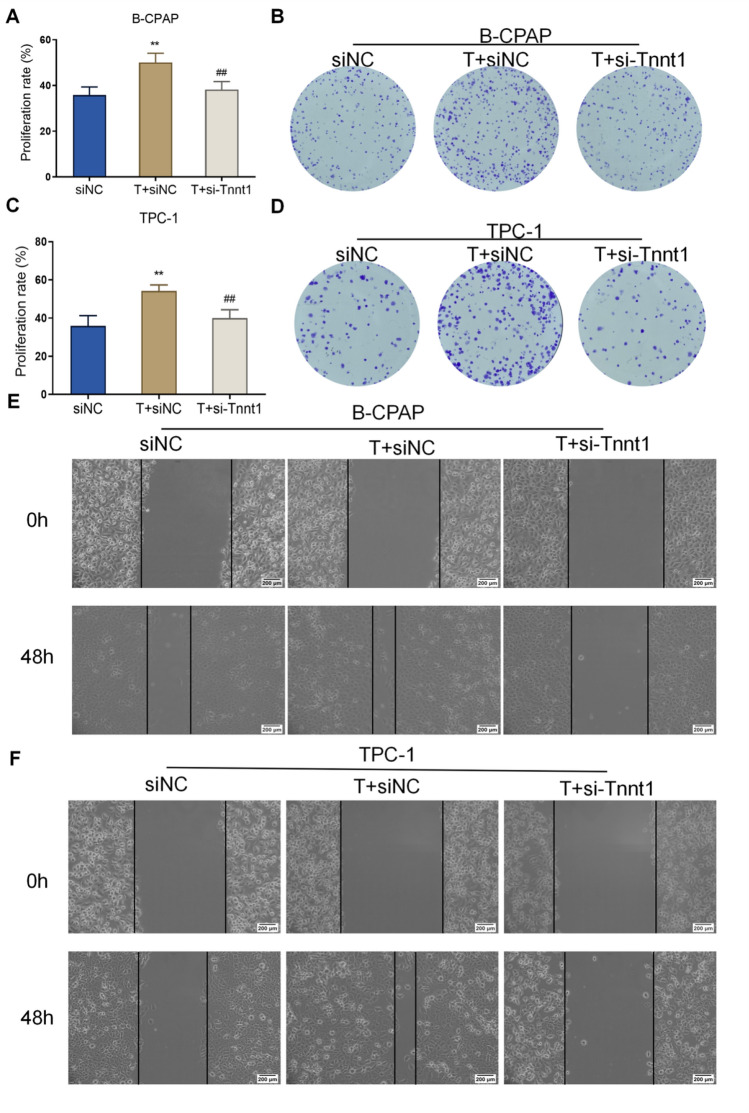
Knockdown of Tnnt1 expression inhibits the proliferation and migration of testosterone-treated PTC cells. The proliferation rate of B-CPAP (**A**) and TPC-1 (**C**) cells after treatment with testosterone alone or that combined with knockdown of Tnnt1 expression checked by CCK-8. The colony formation ability of B-CPAP (**B**) and TPC-1 (**D**) cells after treatment with testosterone alone or that combined with knockdown of Tnnt1 expression determined by colony formation assay. The migration ability of B-CPAP (**E**) and TPC-1 (**F**) cells after testosterone treatment alone or that combined with knockdown of Tnnt1 expression measured by scratch assay. **P < 0.01, vs. siNC, ^#^*P* < 0.05 and ^##^*P* < 0.01, vs. T + siNC

**Fig. 10:**
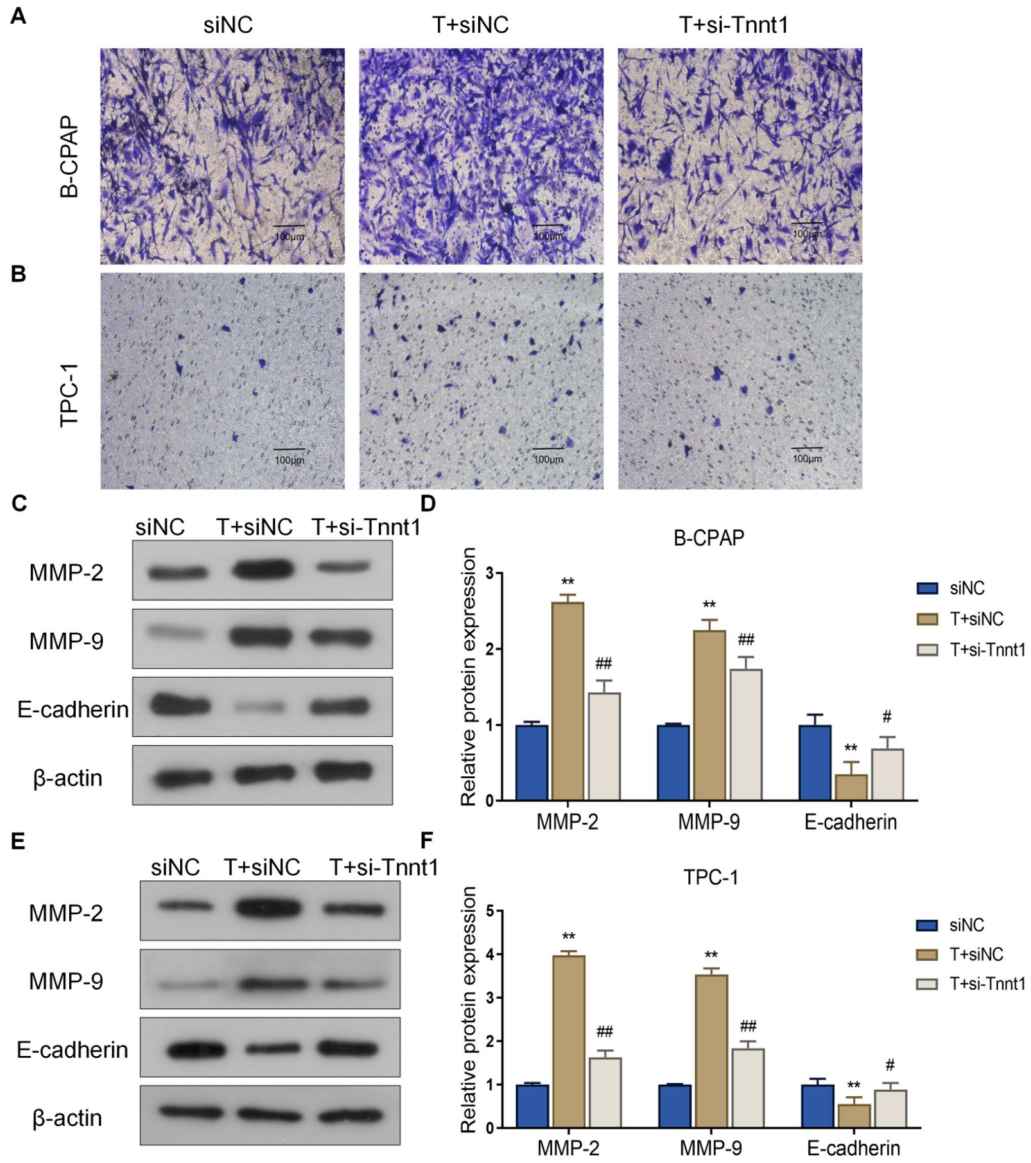
Knockdown of Tnnt1 expression inhibits invasion and EMT progression in testosterone-treated PTC cells. The invasion ability of B-CPAP (**A**) and TPC-1 (**B**) cells after testosterone treatment alone or that combined with knockdown of Tnnt1 expression checked by Transwell. The relative protein expression level of MMP-2, MMP-9 and E-cadherin in B-CPAP (**C**, **D**) and TPC-1 (**E**, **F**) cells after testosterone treatment alone or that combined with knockdown of Tnnt1 expression detected by western blot. **P < 0.01, vs. siNC, ^#^*P* < 0.05 and ^##^*P* < 0.01, vs. T + siNC

**Fig. 11:**
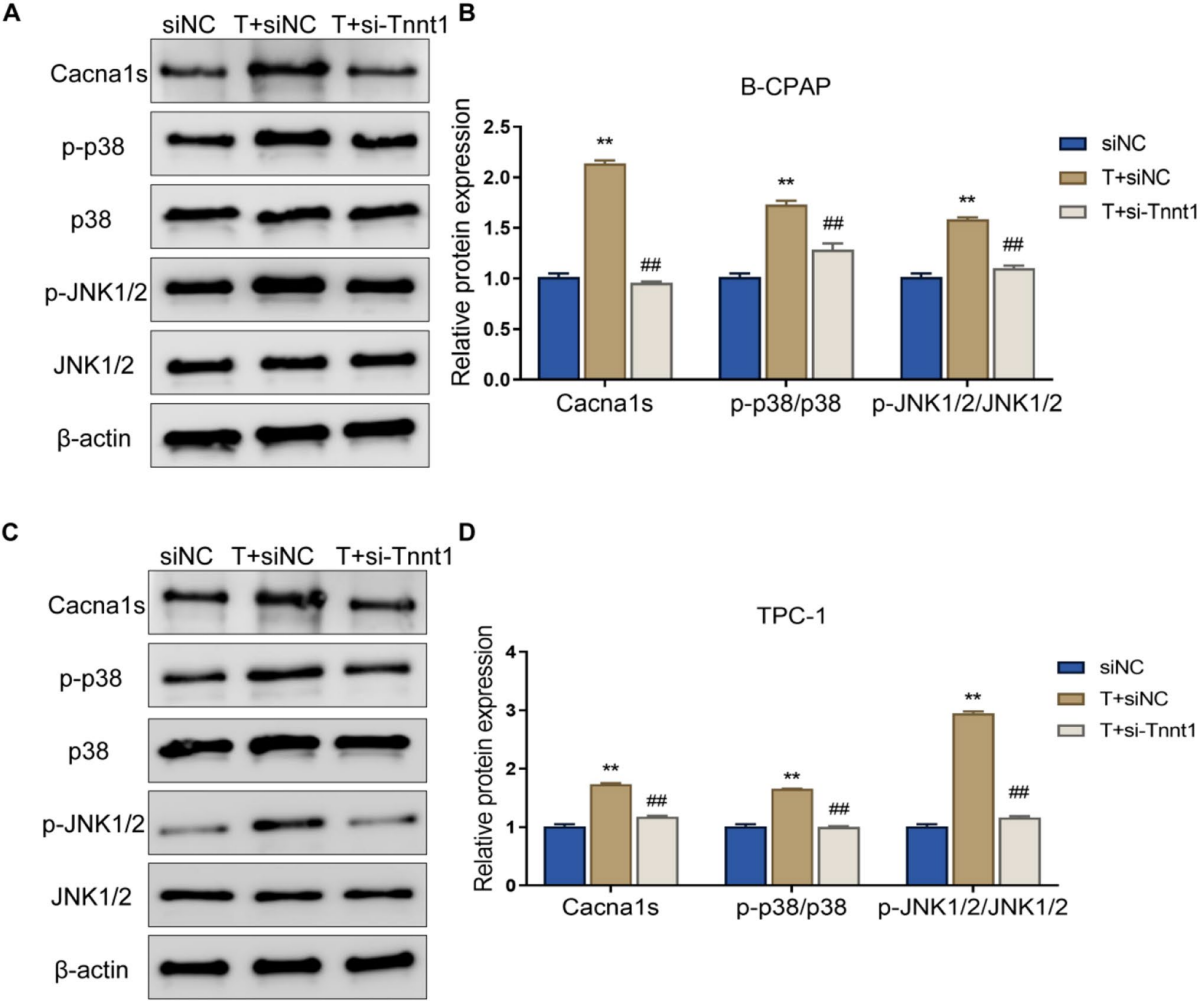
Knockdown of Tnnt1 expression inhibits the p38/JNK signaling pathway in testosterone-treated PTC cells. Relative protein expression level of p38/JNK signaling pathway-related proteins (Cacnals, p-p38, p38, p-JNK, and JNK) in B-CPAP (**A**, **B**) and TPC-1 (**C**, **D**) cells after testosterone treatment alone or that combined with knockdown of Tnnt1 expression tested by western blot, **P < 0.01, vs. siNC, ^#^*P* < 0.05 and ^##^*P* < 0.01, vs. T + siNC

After determining the proliferation, colony formation, migration, and invasion of cells in each group, it was found that the proliferation ability, colony formation ability, migration ability, and invasion ability of PTC cells in the T + siNC group were significantly increased compared with those in the siNC group; while the above cell abilities in the T + si-Tnnt1 group were significantly decreased compared with those in the T + siNC group (Figs. 9A–F and 10A, B). Moreover, western blot disclosed that testosterone alone could notably increase the protein expression level of MMP-2 and MMP-9 and lower the expression level of E-cadherin in the siNC group; after knockdown of Tnnt1 expression, testosterone significantly reduced the level of MMP-2 and MMP-9 and elevated the expression level of E-cadherin (Fig. 10C–F). Further examination of p38/JNK signaling pathway activity revealed that testosterone treatment resulted in a significant increase in p38/JNK signaling pathway activity in PTC cells; while after knockdown of Tnnt1 expression by si-Tnnt1, testosterone obviously inhibited p38/JNK pathway activity (Fig. 11A–D). In summary, testosterone could significantly promote the proliferation, migration, invasion and EMT process of PTC cells and could enhance the activity of p38/JNK signaling pathway. However, if knockdown of Tnnt1 expression, the activation of PTC-induced malignant phenotype and p38/JNK signaling pathway was reversed. It could be concluded that testosterone played a cancer-promoting role by up-regulating Tnnt1 expression.

## Discussion

In this study, we investigated the effect of testosterone on the tumor formation of PTC cells, and the results indicated that both estradiol and testosterone could promote the growth of PTC cell xenografts. To reveal the mechanism of action of testosterone in PTC, the tumor tissues of the testosterone group and Control group were collected for RNA sequencing and bioinformatics analysis. According to analysis outcomes, the main functions of DEGs were associated with a series of muscle activities such as muscle system process, muscle cell differentiation, striated muscle contraction, contractile fiber, proteinaceous extracellular matrix, sarcolemma, actin binding, sulfur cycle binding, channel activity, as well as molecular pathways such as TCA and oxidative phosphorylation. Moreover, we constructed a PPI network of DEGs and screened key genes. The outcomes revealed that genes such as Ttn, Actn2, Myl1, Ldb3, Actn3, Neb, Tcap, Atp2a1, Ckmt2, Ckm, Myl3, Myoz1, Myl2, Mylpf, Acta1, Myh4, Trdn, Cav3, Obscn and Tnnt1 played a key role in the PPI network. In addition, the above genes were also found to be mainly related to myocytes and muscle fibers after the literature review. In a word, the findings of key gene screening by the PPI network were consistent with the results of GO and KEGG functional analysis in this paper.

RNA-Seq sequencing results showed that Tnnt1 expression was significantly up-regulated in PTC mice after ORX and injection with testosterone, indicating that testosterone may promote the development of PTC by up-regulating Tnnt1 expression. Troponin T (TNT), an important protein of about 30–35 kDa containing about 220–300 amino acids, can regulate contraction and relaxation of striated muscle [24]. Troponin T1 (Tnnt1) is a subunit of TNT [25]. Recently, many articles have pointed out that Tnnt1 expression was closely correlated with the development of colorectal cancer and breast cancer. For example, Shi et al. stated that Tnnt1 was significantly highly expressed in breast cancer tissues, and its high expression was closely related to the clinical grade and T and N stages. At the same time, Tnnt1 was discovered to increase the proliferation rate of breast cells by promoting G/S phase transformation [26]. In addition, several studies have disclosed that Tnnt1 expression was significantly increased in colorectal cancer. The high expression then promoted the proliferation, migration, invasion, and EMT process of colorectal cancer cells, which was strongly linked to poor prognosis, higher T stage, differentiation degree, and lymph node metastasis [27, 28]. In this study, Tnnt1, as one of the key genes in the PPI network, displayed observably up-regulated expression in tumors from mice in the Testosterone group (log (FC) = 7, *P* < 0.05). However, the function of Tnnt1 in PTC was not yet explored. We found that overexpression of Tnnt1 notably promoted the proliferation, activity, migration, invasion, and EMT process of PTC cells, while knockdown of Tnnt1 expression showed the opposite results. The above findings suggested that Tnnt1 promoted PTC development, which was consistent with previous studies. In addition, the correlation between testosterone treatment and Tnnt1 expression needs further verification. Through testosterone treatment and knockdown of Tnnt1 expression, we demonstrated that si-Tnt1 was able to effectively inhibit the promoting effect of testosterone on the proliferation, migration, invasion, and EMT of PTC cells as well as PTC growth in vivo. The above outcomes in this study suggested that testosterone could promote the malignant progression of PTC by up-regulating Tnnt1 expression.

Uncertainty exists regarding the molecular mechanism by which Tnnt1 affects the proliferation, migration and invasion of PTC. Mitogen-activated protein kinase (MAPK) signaling pathway can regulate multiple cellular activities involved in cancer progression, including cell proliferation and cell cycle. In addition, c-Jun N-terminal kinases (JNKs) and p38 are the two major molecule hubs in MAPK signaling pathway [29]. Generally speaking, the activated p38 and JNK play complex roles in cancer, and the phosphorylation of p38 and JNK is associated with tumor cell growth. Studies have shown that abnormal active p38/JNK signaling pathway can lead to excessive proliferation and inhibition of apoptosis in cancer cells, which also contributes to the development, migration and invasion of various malignant tumors [30–32]. Chen et al. reported that the p38/JNK signaling pathway can promote tumorigenicity of glioma cells and self-renewal of glioma stem cells (GSC), and this promotion aids in malignant progression and development of glioblastoma [33]. Previous studies demonstrated that inhibition of JNK and p38 pathways can suppress the proliferation of PTC cells [34, 35]. What is more, Cui et al. revealed that JNK and p38 pathway inhibitors, SB225002, SP600125 and SB203580, suppressed the growth of PTC cells in nude mice [36]. These earlier investigations and our findings confirm the significance of the p38 and JNK pathways in PTC. This study was the first to report the conclusion that Tnnt1 could significantly activate p38/JNK signaling pathway activity. Therefore, p38/JNK signaling pathway may be involved in the process where Tnnt1 affects the proliferation, migration and invasion of PTC.

According to the previous findings of this study, the injection of testosterone or estradiol could significantly promote the growth of PTC xenografts in mice. Then, high-throughput RNA sequencing revealed that testosterone treatment caused a significant up-regulation in 2617 genes and a down-regulation in 85 genes in mouse xenografts. After a series of bioinformatics analyses, the key gene Tnnt1 was obtained. Tnnt1 was highly expressed in tumor tissues and PTC cells of testosterone-treated mice. As revealed by functional analyses, overexpression of Tnnt1 not only significantly promoted the proliferation, colony formation, migration, invasion and EMT process of PTC cells, but also activated the p38/JNK signaling pathway. Besides, knockdown of Tnnt1 expression can inhibit the cancer-promoting roles of testosterone in PTC cells. This study demonstrated that testosterone could accelerate the malignant progression of PTC by up-regulating Tnnt1 expression, and the acceleration may be related to its regulation on p38/JNK pathway. However, this study failed to use pathway inhibitors to verify that the testosterone exerts its effect by up-regulating Tnnt1 expression to activate p38/JNK signaling pathway. In addition, only Tnnt1 was examined in subsequent studies, despite the vast number of up-regulated DEGs that were discovered in our investigation. In addition, many potential mechanisms of sex hormones remain unclear. If further studies are conducted, the mechanism by which sex hormones promote PTC progression can be further clarified.

## Conclusion

Overall, testosterone may up-regulate Tnnt1 expression to activate p38/JNK pathway activity, which makes PTC promotion possible. This study provides important experimental and preclinical basis for the early diagnosis and intervention treatment of PTC.

### Supplementary Information

Below is the link to the electronic supplementary material.

Supplementary file1 (DOCX 4435 kb)

## Data Availability

The dataset supporting the conclusions of this article is available from the corresponding author.

## Abbreviations

PTC: Papillary thyroid carcinoma
ORX: Orchiectomy
DEGs: Differentially expressed genes
PPI: Protein–protein interaction
TCA: Tricarboxylic acid cycle
