# Supplementary material for: Testosterone promotes the migration, invasion and EMT process of papillary thyroid carcinoma by up-regulating Tnnt1
Source: J Endocrinol Invest. 2023 Jul 21;47(1):149–66. doi: 10.1007/s40618-023-02132-1 (PMC10776714; doi:10.1007/s40618-023-02132-1)
Supplement: Supplementary file 1 — Supplementary file1 (DOCX 4435 kb) [file 40618_2023_2132_MOESM1_ESM.docx]

**
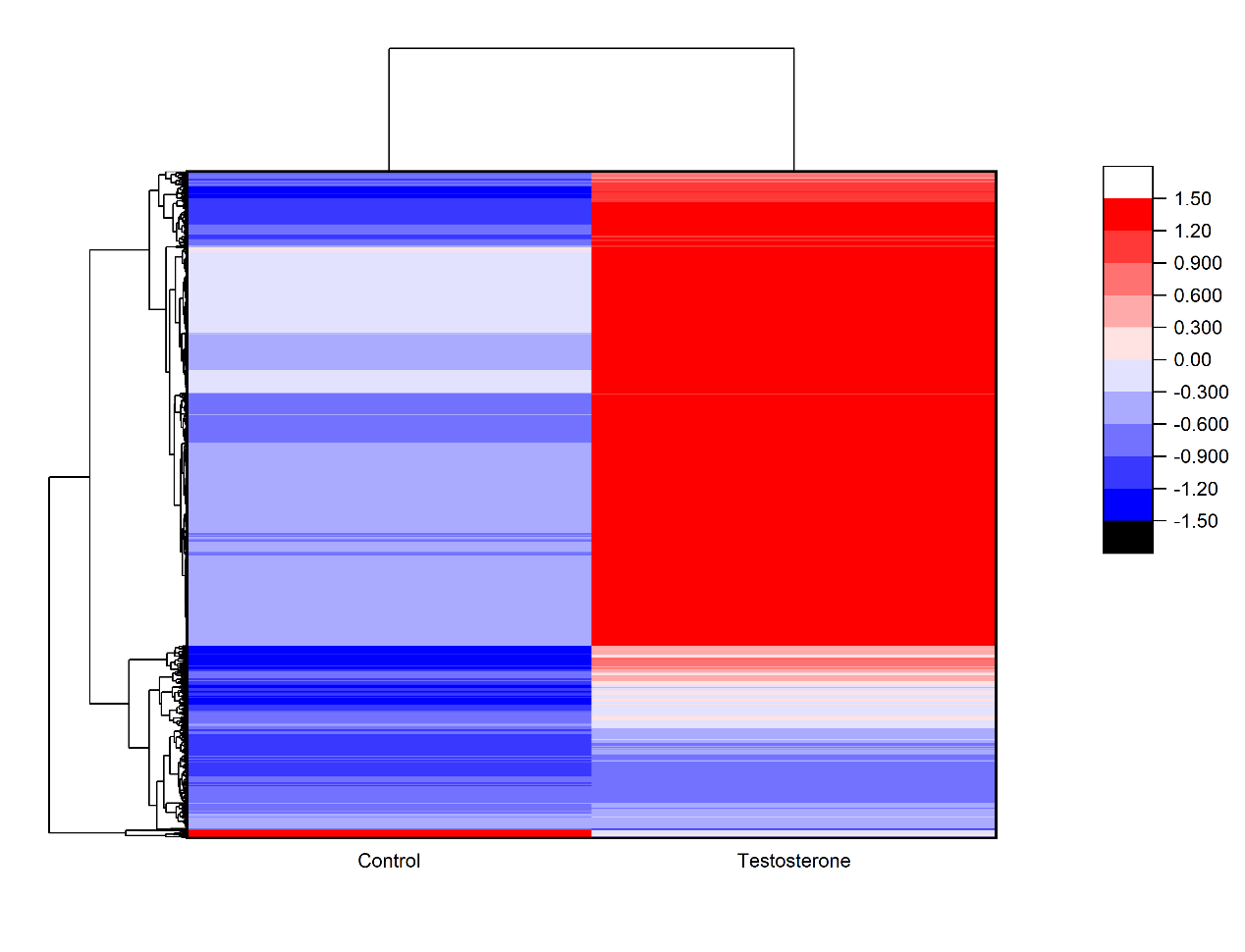
Figure S1** Heat map. The clustering and expression of DEGs in tumor tissues of mice in the Testosterone group and Control group shown by heat map, with blue representing the down-regulated genes. and red representing the up-regulated genes.


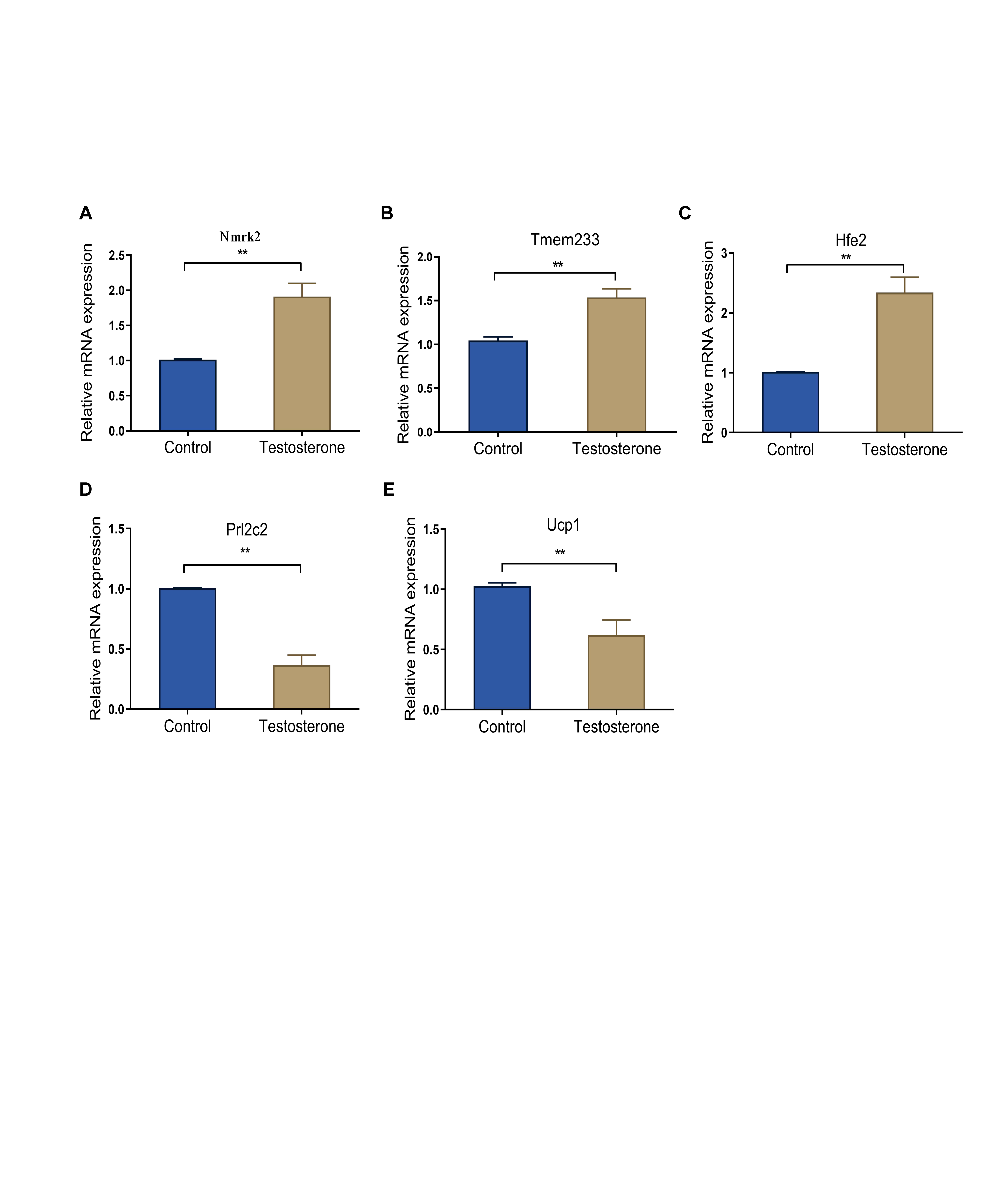


**Figure S2** Differentially expressed genes validated by qRT-PCR. A-E: The mRNA expression level of Nmrk2, Tmem233, Hfe2, Prl2c2 and Ucp1 in the tumor tissues of mice in the Control group and Testosterone groups detected by qRT-PCR, *P<0.05 and **P<0.01, vs Control.

**Table S1** The results of the high-throughput sequencing of the Nmrk2, Tmem233, Hfe2, Prl2c2 and Ucp1.

| ensembl_gene_id | log2FoldChange | P value | Adjust P value | Style | external_gene_name | gene_biotype | Control | Testosterone |
| --- | --- | --- | --- | --- | --- | --- | --- | --- |
| ENSMUSG00000004939 | 14.12018963 | 0.000242329 | 0.007621437 | up | Nmrk2 | protein_coding | 0 | 69.91460221 |
| ENSMUSG00000079278 | 13.96659365 | 0.00023591 | 0.007450967 | up | Tmem233 | protein_coding | 0 | 47.04492721 |
| ENSMUSG00000038403 | 13.6959063 | 5.95E-12 | 1.27E-09 | up | Hfe2 | protein_coding | 0.009770743 | 129.5002386 |
| ENSMUSG00000079092 | -7.151014518 | 0.00845537 | 0.120793843 | down | Prl2c2 | protein_coding | 1.30468456 | 0 |
| ENSMUSG00000031710 | -6.331029791 | 0.009928473 | 0.135980614 | down | Ucp1 | protein_coding | 0.498100263 | 0.023672669 |
